# The Future of Laser Acupuncture—Robot-Assisted Laser Stimulation and Evaluation

**DOI:** 10.3390/life13010096

**Published:** 2022-12-29

**Authors:** Gerhard Litscher

**Affiliations:** Research Unit of Biomedical Engineering in Anesthesia and Intensive Care Medicine, Research Unit for Complementary and Integrative Laser Medicine, Traditional Chinese Medicine (TCM) Research Center Graz, Department of Anesthesiology and Intensive Care Medicine, Medical University of Graz, 8036 Graz, Austria; gerhard.litscher@medunigraz.at; Tel.: +43-316-385-83907

**Keywords:** laser acupuncture, laser stimulation, computer-controlled acupuncture, robot-assisted laser acupuncture, smartphone, home treatment, photobiomodulation

## Abstract

This brief contribution is part of a Special Issue entitled ‘Laser Acupuncture: Past, Present and Future’ and primarily deals with the future of laser acupuncture from the author’s perspective. The procedure from developing the first laser to robot-assisted laser acupuncture is briefly shown. The latter has already become a reality and, in the near future, will be made accessible to a broad group of patients as a home treatment system developed by researchers from Taiwan. The new equipment is based on a smartphone with integrated artificial intelligence methods (e.g., automatic image recognition).

## 1. Introduction

The first laser came into being in 1960 [1]. The history of laser acupuncture (LA) began shortly thereafter. This history was recently summarized in a review article in a comprehensive form [2]. Within the scope of this editorial, a prediction and speculations are made as to how LA could develop further as an independent method and as a procedure in combination with so-called photobiomodulation (PBM) techniques in integrative medicine [3]. 

## 2. Definition of Laser Acupuncture

The number of LA studies listed in the Science Citation Index (SCI) and PubMed databases is steadily increasing. In Pubmed, there are 1188 articles on this topic as of 22 December 2022. The approved definition of LA and all kinds of photo acupuncture is: “Photonic stimulation of acupuncture points and areas to initiate therapeutic effects similar to that of needle acupuncture and related therapies together with the benefits of PBM” [4].

## 3. State of the Art and Future Aspects of Laser Acupuncture Stimulation

### 3.1. State of the Art

A repeated question regarding LA is that it only has limited stimulation methods. With manual needle acupuncture, for example, you can influence different stimulation modalities (lifting and thrusting the needle or rotating techniques), which should more complex withLA. But that is not true. Quite the contrary, with LA, there have recently been more stimulation options than with needle acupuncture.

These stimulation techniques are as follows:

Continuous wave stimulation.

Different frequencies.

Changing the focal point of the laser.

The most commonly used method is laser stimulation (continuous wave mode; cw). Most of the scientific articles concern laser cw stimulation. The wavelengths used in this context are from the red and near-infrared light ranges. However, many other wavelengths are also used for acupuncture. For example, a violet, blue, green or yellow light or laser is also used; there are numerous relevant scientific publications [1].

In addition, the LA provides the possibility of stimulating with different frequencies. This has been studied, but it must be mentioned that many of the frequencies used in praxis (Nogier, Bahr, Reininger, and others) were found exclusively empirically and, according to many authors, cannot be reconciled with an evidence-based method. Nothing has changed since Robinson’s critical statement eight years ago [5]. Of course, individual experiments [6] and comments refer to the different frequencies. However, as mentioned before, according to scientific criteria, these frequencies have been found empirically, are based on some doctors’ experience, and should only be used with great caution in evidence-based therapy [5].

Changing the focal point of the laser is a new lift-thrust operation in LA. This method could bring about a significant improvement in the measured and obtained effects of LA. Scientific studies have already proven the initial evidence with a multiplication of effects [7]. Currently, the method researchers from Taiwan developed is in the testing stage, and new data are available almost every month [8].

### 3.2. Future Aspects

In addition to the new laser stimulation techniques mentioned [9], LA’s future lies in automated acupuncture point detection. There is already much scientific work on this, especially from colleagues from Taiwan [8,10]. By integrating machine learning algorithms and computer visualization techniques, robotic acupuncture systems are being developed for needle acupuncture or massage [9,11] and LA [8]. A recently published article describes such a system in more detail [8]. In the near future, this smartphone-based equipment could be used automatically as a home treatment.

Another recent development is that the laser beam is reflected by a galvo mirror and irradiates an object (hand, leg, etc.) [12]. A camera is used to localize human acupuncture points, and by controlling the rotation angle of the galvo mirror, the laser beam is guided to stimulate acupoints. Rotation of the galvo mirror enables multiple points to be treated rapidly and sequentially without the need to reposition a patient or an extremity [12].

## 4. Discussion and Conclusions

As early as 1997, our research team at the Medical Faculty of the University of Graz in Austria could demonstrate that acupuncture also works without the manual intervention of an acupuncturist. At that time, we created the term ‘Computer-Controlled Acupuncture’ [13,14]. It was never our intention that the computer should replace the human and, thus, the acupuncturist. Instead, we tried for the first time to register the quantifiable effects of acupuncture. The LA was an irreplaceable tool for us. For the first time, real double-blind studies could be carried out, and evidence of the specific effect of LA on the brain was also obtained [1]. We also created the term ‘Computer-Controlled Laser Acupuncture’ [15]. However, it took 25 years for the vision of robotic LA to become a reality.

Researchers from Taiwan have now realized this [10], and we are, of course, pleased that we were partly involved in the publication of this interesting research work. ‘Robot-Assisted Laser Acupuncture’ is no longer an abstract expression; as mentioned, it has become a reality in 2022. The hope is that it will be possible to implement this on a smartphone as a home treatment option so that many patients can have direct help.

## Data Availability

Data supporting reported results can be found by the author.
